# Ambient Teens Sleep Study: Protocol for a co-produced feasibility study in adolescents using a contactless radar-based sleep sensor

**DOI:** 10.1371/journal.pone.0313286

**Published:** 2024-11-14

**Authors:** Lorna Caddick, Giulia Gaggioni, Dawn Haughton, Judith Brown, Joanna Inchley, Sharon Anne Simpson, Laura M. Lyall, Aja Murray, Liana Romaniuk, Lukas Krondorf, Lorna M. Lopez, Daniel J. Smith, Cathy Wyse, Heather Whalley

**Affiliations:** 1 Division of Psychiatry, Centre for Clinical Brain Sciences, University of Edinburgh, Edinburgh, United Kingdom; 2 Medical Research Council/Chief Scientist Office Social and Public Health Sciences Unit, University of Glasgow, Glasgow, United Kingdom; 3 Institute of Health and Wellbeing, Mental Health and Wellbeing, University of Glasgow, Glasgow, United Kingdom; 4 Department of Psychology, University of Edinburgh, Edinburgh, United Kingdom; 5 Department of Data Science and Research, Vitalthings AS, Trondheim, Norway; 6 Department of Biology, Kathleen Lonsdale Institute for Human Health Research, Maynooth University, Maynooth, Co. Kildare, Ireland; 7 Institute of Genetics and Cancer, Generation Scotland, University of Edinburgh, Western General Hospital, Edinburgh, United Kingdom; PLOS: Public Library of Science, UNITED KINGDOM OF GREAT BRITAIN AND NORTHERN IRELAND

## Abstract

Sleep is crucial for the healthy development of adolescents, yet many suffer from chronic sleep deprivation. Over the transition to and course of adolescence there are known changes to sleep patterns e.g. shifts towards evening chronotypes. To study changes and patterns of sleep over these critical developmental time-points, detailed longitudinal data is required over months/years rather than weeks/days, a typical limitation of current technology. The implementation of novel contactless sleep sensors offers significant opportunities for longer term data collection, but their application has yet to be explored in young people in terms of feasibility, acceptability, performance and operability. The Ambient Teens Sleep Study will assess the feasibility of a contactless sleep sensor with approximately 45 adolescents aged 8–18 years, for 4 weeks. The device will be compared with accelerometery and sleep diary data, collected concurrently (2 weeks). Young people will provide feedback in the form of online calls and questionnaires as well as citizen scientist and product reviewer activities. Baseline questionnaires, conducted online, will be used to determine acceptability in different cohorts such as age, sex, gender and geographic location. We aim to assess completeness of data, participant experience and performance of sleep timing measures across all three methods of sleep assessment (contactless radar, accelerometery and sleep diary). The results will be used to inform longitudinal methods of sleep data collection in future adolescent health studies, at scale, to explore links between sleep and essential health outcomes across adolescent development.

## Introduction

Sleep habits are known to dramatically change over the course of childhood, adolescence and early adulthood [1]. Around 50% of young people living in industrialised societies experience chronic sleep deprivation, influenced by physiological, lifestyle and environmental factors [2]. The gold standard method for assessment of sleep is polysomnography [3], which is a cumbersome and expensive process that typically requires a sleep laboratory. Sleep can also be assessed using subjective measures (diaries or questionnaires) [4] or using wrist-worn accelerometers (actigraphy) usually over study durations of 1–2 weeks [5,6]. The participant burden of accelerometery is not conductive to prospective studies and as a consequence of insufficient longitudinal sleep data, changes to sleep during the critical developmental period of adolescence and links to health outcomes are yet to be fully explored.

Initiated by the onset of puberty, commonly between the ages of 8–14 in girls and 9–15 in boys [7], children experience changes to their regulation of sleep, such as delayed sleep onset [8,9], shift to a later chronotype [10], slower build-up of sleep pressure [11] and a significant reduction in slow wave sleep [12]. Later sleep times without changes to school start times, combined with other lifestyle factors (e.g. evening screen-time, energy drink consumption and insufficient daylight exposure), often result in inadequate sleep quantity and quality [13]. Furthermore, desynchrony between personal chronotype and school start times presents as social jetlag with later bedtime, later wake time and longer sleep durations on weekends compared to weekdays [14,15]. Disrupted sleep starting at a young age is harmful to development and is associated with the onset of physical and mental illnesses that can continue into adulthood [16]. Sleep deprivation during development is associated with reduced cognitive performance and poor school grades [17], increased likelihood of risk-taking during adolescence [18,19], reduced brain size and altered brain structures [20] as well as links with cardiometabolic health [21], obesity [6] and poor mental health [22]. In addition to the biological impact of puberty on adolescent’s sleep, increased social and academic pressures can trigger symptoms of anxiety and peer pressure that may negatively affect sleep, creating a bidirectional relationship between sleep and health [23].

There is growing interest in better understanding adolescent sleep habits to identify patterns to predict and prevent future health complications and support positive health outcomes. Accurate tools to collect longitudinal sleep data in large populations of adolescents are required. Polysomnography may be too invasive for acceptable use in young people and is not easily applied over long periods. Reliable, objective and less invasive wearable devices (including wrist-based accelerometery [24], headpieces [25] and rings [26]) may be viewed as standard for use in young people [27]. However, each method listed requires daily manual input or physical contact that hampers long-term use and engagement. Often wearable devices are only tolerated for up to two weeks due to discomfort, irritation, wearing in addition to their own device and battery limitations [28], restricting accurate representation of typical weekday and weekend life. Contactless sleep sensors that use radar, sonar or lidar technology, grouped as ‘nearables’ rather than ‘wearables’ offer promising new options. Several nearable devices have become available for consumer-measurement of sleep over the last few years, most notably smart speakers and home assistants such as Google Nest Hub and Amazon Halo Rise. However, there is limited information on the performance of these devices and how the parameters they report are derived. Several radar sleep sensing devices have been validated against polysomnography: Sleepscoremax [29] and Somnofy [30], but there is limited information on their application in research or clinical settings.

During radar sleep-sensing, a small device is placed at the individual’s bedside that requires no manual input after installation. The radar sensor emits electromagnetic pulses through which whole-body movement can be detected, allowing estimation of respiratory rate and nocturnal sleep patterns [30]. Radar sleep sensing has been developed for use in adults and no studies have yet taken advantage of the capacity of these unobtrusive devices to collect data over extended time periods. Therefore, studies assessing longer-term (>2 weeks) application, and in adolescents, are required. Contactless assessment of sleep may be a useful tool for collection of high-quality longitudinal sleep data with minimal disruption to daily living, allowing sufficient duration to encompass weekends, holidays, school term, stress etc.,—data that represents the normal life of each individual adolescent. However, the use of radar technology and cloud-based data acquisition raises legitimate concerns about data protection and privacy for adolescents and their families. Concerns about how radar sensors acquire data on sleep and environment activities, such as sound and movement, need to be addressed to establish acceptability of this technology. Access to accurate sleep data over long durations (weeks, months, years) at a population-level is a fundamental requirement for understanding the complex relationships between sleep, adolescent development and associated health and social factors.

To enhance feasibility and engagement with an adolescent population (8–18 years), it is important to consult those in the target age-groups. Involving young people in research topics that affect them brings many benefits to research, wider society and the young people themselves. Additionally, it is in-line with the UN Convention on the Rights of the Child that those under 18 have the right to express their opinion and be heard in matters that concern them [31]. Research stakeholders have also recognised the importance of co-production as it is increasingly becoming a requirement for attaining research funding. Involving young people in the early stages of research [32] can help to pre-empt problems and build a protocol that addresses issues of importance to them. Previous research has shown many positive outcomes of youth-enabled research, helping to create supportive communities, opportunities to learn and grow and provide meaningful benefit across multiple domains of research [33]. Consulting adolescents to determine appropriate real-life scenarios supports ecologically valid research methods [34], which are widely accepted and encourage high participation and retention rates [35] for long-term studies.

This study primarily aims to test the feasibility, acceptability and utility of a novel contactless sleep data collection method in young people (8–18 years). Secondary study objectives are:

1. Assess age-specific acceptability and develop age-appropriate instructional materials.
2. Include young people at all stages of the study to produce data that represents the needs and issues of this age group, including engagement activities and co-production of methods, analyses and dissemination.
3. Compare sleep assessment methods using subjective and objective data collected from the contactless sleep sensor, accelerometer and sleep diary.

## Methods and analyses

### Procedure

The study will be completed in two phases: young person advisory group (YPAG) co-production (see patient and public involvement section), followed by the main study involving four weeks of sleep assessment.

### Participants

Approximately 45 adolescents aged 8–18 years are expected to participate in the study, split into age brackets 8–11, 12–15 and 16–18 years. Participants will be recruited with the help of the Scottish Schools Health and Wellbeing Improvement Research Network (SHINE). SHINE will help to recruit schools and young people from marginalised backgrounds or remote areas with their national reach across all 32 local Scottish authority areas, encompassing a wide variety of socio-economic and urban/rural settings. Recruitment will focus on links with schools and community organisations through SHINE network events, posters and newsletters, as well as utilising social media to engage young people. Participants will be recruited continuously so that 6 contactless sleep sensors can be used in rotation over the duration of the study. Recruitment started on the 11/01/2024 and is expected to continue until 31/12/2024.

### Inclusion and exclusion criteria

Inclusion criteria: aged 8–18 years, fluent in English, living in Scotland, ability to provide informed assent, and caregiver informed consent if <16 years. The young person must have the ability and willingness to install the contactless sleep device and wear the wrist-based accelerometer. There will be no specific exclusion criteria applied to assess the feasibility and usability in adolescents with a range of needs. Research materials will be made accessible, such as age-appropriate versions, video instructions with on-screen captions, and clear contact information to support young people with additional needs. Accessibility funds are available for young people that do not have access to phones and internet services or request additional requirements within reason to promote inclusion.

### Patient and public involvement

The study protocol has been shaped by feedback from young people. YPAGs were formed to advise on the study design and co-produce research materials that are age appropriate and engaging. Members were recruited by distributing posters, SHINE newsletters and information sharing at conferences. Four young people were assigned to each age group (8–11, 12–14 and 15–18 years). For members under 16, their caregivers were also invited to attend YPAG sessions.

Each group held 2–3 online sessions (Microsoft Teams) that included sleep education, overview of the study and Q&A sessions through an anonymous online platform (Wooclap or Mentimeter). Members aged 15–18 years trialled the contactless sleep sensor and gave short presentations on their experience. This feedback has guided the design and wording of instructions in written and video formats. Other examples of major changes based on YPAG discussions include: weekly check-ins to provide structured communication between participants and researchers and share summary sleep data for young person engagement, generation of ideas for the product reviewer and citizen scientist activities, as well as approaches for recruitment and retention of other young participants.

All YPAG members were offered certificates of participation, thank you letters and a £20 voucher for their time and involvement. Young people will continue to shape the project with opportunities to contribute to the co-production of analysis, report results as co-authors and advise the best approaches for dissemination to young audiences.

### Data collection

**Fig 1:**
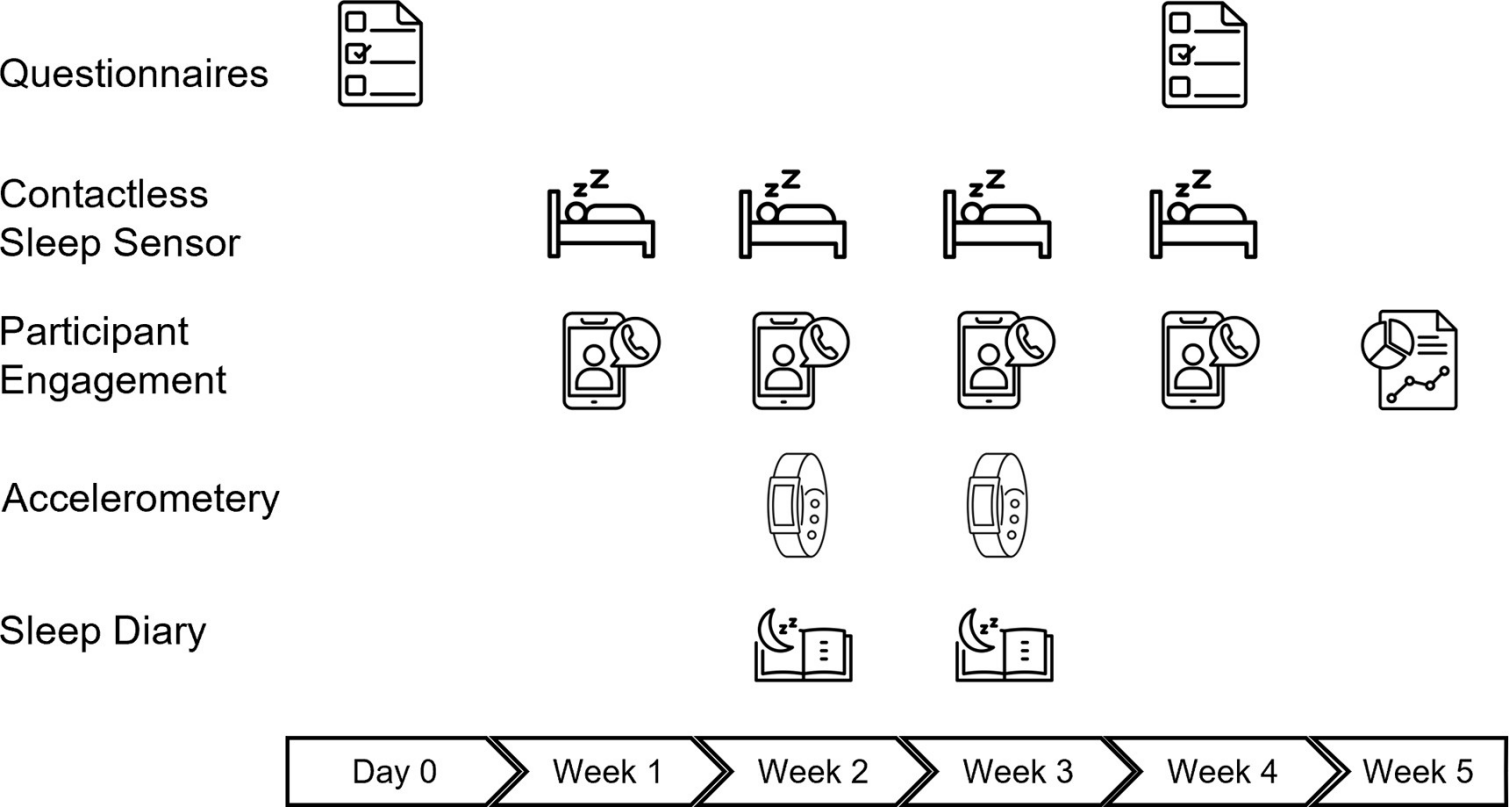
Summary of Ambient Teens data collection for each individual participant. Multimodal data acquisition includes contactless sleep sensor (4 weeks), accelerometery (2 weeks), and sleep diary (2 weeks). Questionnaires include consent form, demographics survey, PDS, RCADS, MCTQ, and SDSC at baseline followed by a feedback questionnaire at the end of collecting sleep data. Participant engagement includes weekly check-ins and product reviewer/citizen scientist activities. PDS: Pubertal Development Scale, RCADS: Revised Children’s Anxiety and Depression Scale, MCTQ: Munich ChronoType Questionnaire, SDSC: Sleep Disturbance Scale for Children. All icons are designed by Freepik.

Data collection started in January 2024 and is estimated to take one year to complete. Fig 1 summarises the study flow.

Following written informed consent, demographic and baseline questionnaires are completed by participants: Revised Children’s Anxiety and Depression Scale (RCADS) [36], Pubertal Development Scale (PDS) [37], Munich Chronotype Questionnaire (MCTQ) [38,39] and Sleep Disturbances Scale for Children (SDSC) [40]). If participants are < 16 years, written caregiver consent is required and RCADS, PDS, SDSC and demographic questionnaires are caregiver-reported. Online Surveys (version 3) will be used to deliver the questionnaires.

The contactless device (Somnofy SM-100) will be sent to the participants home or school with age-appropriate instructions (written and video) demonstrating how to set-up the sleep monitor and WiFi modem near their bed, to be used for 4 weeks. Support with setup will be provided by the research team via a dedicated study phone and email. The young person’s caregiver will also be advised to aid installation of the device. Sleep monitors will be pre-configured with WiFi modems by the research team to minimise participant burden. Audio and light device settings have been discussed with YPAG members to provide the least sensory distraction for sleeping while ensuring feedback that the device is working. The approach to the hours of sleep tracking will be flexible, with the option to disable daytime hours specific to each participant’s sleep times. Additional items such as mounting options, extension cables and dual USB-plugs will be provided based on the needs of an individual. Once set up, the young person is instructed to measure the distance between the device and the far edge of the bed and send this measurement to the research team to adjust the device settings to the most appropriate radar range. Once the device has been installed there should be no further input required by the young person. The research team will check the online admin system (participants do not have access to this) to ensure high-quality data is being collected and will communicate with the participant if there are any problems identified, to change the positioning or connections of the device. A reporting procedure to record issues with setup and equipment will be implemented to collate feedback and assess feasibility.

Concurrently, for two weeks (middle weeks to reduce information overload), the young person will be asked to wear an accelerometer (Axivity AX3) on their non-dominant wrist and complete a hand-written sleep diary [41]. This will be delivered to the participants home or school alongside written wear and care instructions. The activity watches will be fully charged and initialised by the research team.

During data collection, young people, and their caregivers, will be invited to a one-to-one online weekly check-in call. These will allow any questions or queries to be answered and to encourage engagement via sharing small amounts of the participants own summary sleep data.

At the end of the data collection period, the participant will be asked to package up all of the equipment (in pre-paid packaging) and a collection date will be arranged. The participants will then be asked to provide feedback via an online questionnaire. This will include open-text box questions on both devices (Somnofy and Axivity) as well as their overall experience with the study protocol.

Following sleep data collection, participants will be asked to complete an optional engagement activity as a product reviewer (comparing the contactless device with the accelerometer and sleep diary) or citizen scientist (creating their own research question and report using their own, or template, sleep data). The young person will be given guidance for the task but is free to choose their own method of completion, e.g. creative poster, blog post for the study website, video or voice recording. Completed tasks will be invited to be published on the study website with the choice to stay anonymous.

All participants will be offered graded incentives based on their completion of the project, including a voucher up to £30, a thank you letter, certificate and sleep summary. Young people will also be invited to take part in the co-production of analysis and reporting of results as co-authors for articles to be disseminated to young audiences.

### Data processing

Contactless radar-sensor–Somnofy SM-100: high-resolution movement/respiratory data collected on the device will be uploaded to the Somnofy Cloud for algorithmic processing. Additional indices of sleep quality and duration (1-day resolution) are provided together with environmental data: air quality, temperature, light and sound level. In-house algorithms may be used for further data processing.

Accelerometer—Axivity AX3: will be configured to record raw acceleration data at a frequency of 50Hz. Data will be downloaded using the open-source Open Movement GUI software at the end of the acquisition. Accelerometer data will be analysed using free software such as GGIR or UK Biobank accelerometer analysis packages and variables of interest computed.

Sleep diary: returned paper copies will be scanned and compared with objective measures and questionnaire answers.

Questionnaires: Demographics (22-items), Feedback (24-items), PDS (5-items; [37]), RCADS (47-items; [36]), SDSC (26-items; [40]), MCTQ (33-items; [38,39]), will be scored following reference paper guidance and free text responses summarised.

### Measures

The primary focus of this study is to assess feasibility and acceptability of a contactless sleep sensor. From weekly check-in calls and end-of-study feedback questionnaire, participant experience such as ease of use, comfort, support requirements, influence on daily activities and impact on sleep will be summarised for future recommendations. We aim to identify obstacles to data collection in a young population and offer solutions for future work.

Complete data exports for 2–4 weeks of nightly sessions from the contactless sensor, accelerometer and sleep diary will be available to conduct in-depth exploratory analysis of sleep parameters. Measures of interest include sleep onset, sleep offset, sleep midpoint and total sleep duration to be compared between all three methods of sleep assessment. Other measures such as the influence of environmental data (e.g. light, sound, temperature) and subjective responses may also be explored against sleep parameters.

### Analytical procedure

To assess the feasibility and acceptability of the sleep sensors and engagement of young people with the study, initial analyses will assess completeness of data, dropout rates and subjective feedback questionnaire answers. More specifically, demographic and baseline data will be used to determine if there are biases in completeness of data in relation to specific cohorts such as deprivation level (SIMD), geographical area (rural vs urban), age, sex or gender. The influence of contactless sensor positioning across different bedroom arrangements will be assessed using participant feedback and measures such as mean distance between the sensor and participant. Analysis will include subjective and objective comparison using available data based on adherence to all three sleep assessment approaches: contactless radar-based sleep sensor (Somnofy), actigraphy (Axivity AX3) and sleep diary [41]. Guided by participant engagement and co-production, preliminary, exploratory correlations between sleep measures and, for example, chronotype, anxiety/depression scores, pubertal stage or ambient data (light and sound level) may be analysed.

Bland-Altman statistics will assess concordance of sleep duration and sleep timings between objective and subjective measures. Linear mixed-effects models will be applied to assess if sleep measures (averaged over the acquisition period) will vary depending on the factor “age group” (8–11 vs 12–15 vs 16–18). Individual intercepts will be included as a random effect and covariates will be sex, SIMD, rural vs urban.

### Sample size and study duration

The sample size (45 individuals and 28 nights) was selected based on previous radar sleep sensing validation studies [14,28,42,43]. The longitudinal design, limited access to sleep sensors and short funding window, required a resource constraint approach [44] to be applied. However, our selection of sample size (n = 45) ensures a diverse sample to check feasibility across age ranges, genders and other demographics, whilst being logistically achievable. A power calculation was used to determine the minimum correlation that can be detected with 80% power and alpha = 0.05, is r = 0.404. Studies using wrist-based accelerometery are often limited to two weeks of data collection due to the battery life of the devices [45]. In order to test duration limitations of the contactless sleep device, the study is designed to extend beyond the typical 2 weeks of studies using wearable devices. Four weeks was selected to ensure the rotation of six devices would achieve the target number of participants within the study timeframe. A subgroup of participants may collect data for up to 8 weeks to determine feasibility beyond 4 weeks, providing insights of extended data collection.

### Ethics

Sponsorship was authorised by ACCORD on 20/10/2023 (REF: AC23150) before submission of study documents for ethical approval of data collection by the Edinburgh Medical School Research Ethics Committee. The Ambient-Teens project received a favourable ethical opinion on 11/01/24 (REF: 23-EMREC-053). Additional information regarding the ethical, cultural, and scientific considerations specific to inclusivity in global research is included in the (S1 Checklist).

## Discussion

This protocol outlines the intended methods for a co-produced feasibility study exploring sleep assessment techniques in adolescents. The results from this study will inform best practice use of ecologically valid sleep assessment in young people’s homes. Guidance on conducting sleep assessment studies using radar sensors in adolescents, informed by young people, will be developed and made widely accessible.

The target age range of this study (8–18 years) is intended to gather feedback from young people at all stages of adolescent development. As children develop into young adults, maturation of the prefrontal cortex encourages independent decision making and confidence in personal preferences [46]. Hence, it is important to seek responses regarding acceptance of sleep-sensing devices from all ages, which in turn is likely to influence the levels of support required. Consequently, as children grow up, their bedrooms often change with them. Transition from shared bedrooms, bunkbeds, and bed designs such as raised or cabin beds, progresses to favoured personal space with individual bedrooms and introduction of double beds [47]. The capacity for families to provide individual bedrooms for each child is also largely impacted by socio-economic status [48]. Additionally, the current generation of children and young people have been exposed to increased screen-use [49] leading to more time spent relaxing in their bedrooms rather than socializing in other areas of the home. These observations are important to contextualise data and distinguish between time spent in bed and time spent asleep to accurately explore relationships with health and wellbeing. Significant changes to bedroom arrangements and behavioural lifestyle choices as individuals get older are expected to have a large impact on the acceptance and feasibility of sleep-sensing devices, supporting the importance of conducting this work in the target age range.

To encourage adherence to long-term data collection, adolescent engagement and low maintenance solutions are of key importance. Many find it difficult to wear technology in bed, reporting discomfort as well as battery-life limitations [15] and so opting to place wearables on their bedside table or charge overnight. Other negative experiences have been reported [50] including skin irritation and remembering to remove and put back on wearable devices after water-based activities. Currently, in research settings, minimising participant burden has been approached by reducing the duration of wearing actigraphy monitors, sacrificing longitudinal data. Collecting long term data (>2 weeks) to analyse changes across significant life events in an adolescent population is now more accessible due to the development of novel contactless sleep sensors. The current study aims to address concerns about radar-based sleep technology, seeking evidence that devices are safe, unobtrusive and user-friendly, and develop resources required to implement the use of these devices in the bedrooms of young people from a diverse range of backgrounds. Contactless sleep sensors offer solutions to many of the challenges presented by wearable devices and subjective methods to assess sleep, such as a direct source of power and no manual data entry, as well as other research benefits including real-time data uploads. Taking steps to examine the acceptability of this technology is vital for use in a scalable research capacity.

It has not previously been possible to collect objective continuous sleep data over months and years. The results of this study will support scaling for future longitudinal cohort studies to explore sleep in adolescents at a much greater depth. This can include better understanding of normal sleep for age or development stage, as well as the influence of disrupted sleep on physical health and psychopathologies. Sleep is a potentially modifiable risk factor that, with well-informed best practices, can be optimised to reduce the consequences of disrupted sleep and circadian rhythms. Targeting these issues in the younger generation supports research findings that can protect against early onset symptoms indicative of future health problems.

Our use of co-production is invaluable to engage young people in longitudinal research studies. Important research decisions to consult young people include: regularity of communication, formats of instructions, age-appropriate wording and how to advertise to the target age groups [33]. Examples from current work with young people suggests wording of support materials needs to be aimed and adjusted for target age groups (8–11, 12–15 and 16–18 years). Terms such as “bedtime” can be viewed as childish for older participants who want to receive education about specialist sleep terms and be challenged in their understanding. Contrastingly, the youngest age-group require information broken down into basic terms with additional versions for caregivers. We aim to continue working closely with our young person advisors to disseminate findings and promote sleep education across Scotland.

### Limitations

The use of radar technology for the assessment of sleep is a relatively new concept. In turn, there is limited research [14] to support methodological decisions, particularly in participants as young as 8-years-old. Examples of challenges that need to be addressed include: positioning of devices integrated within the sleeping arrangements of young people (e.g. bunk beds and shared rooms), the duration of time young people spend in their bedroom [51] that can be detected by the device, and the influence of pets in bedrooms. Furthermore, in school-settings, following advertisement and registering interest, teachers select who will take part. These adolescents may be more engaged than the average adolescent and so this sample may not represent the wider population. Thus, this study will identify areas and issues that should be addressed to encourage wider acceptability and availability of radar technology for sleep assessment.

### Dissemination

Anonymous data will be deposited in an open access repository according to the UK Data Service and Open Science Framework. Results will be disseminated via publication, conference presentation and reporting on the study website. Young people will also be invited to co-write articles for the dissemination of project outcomes to target young audiences. We will offer to present findings to stakeholders via school assemblies and webinars. The public will continue to be engaged through media appearances, public talks, science-festivals and school-based events. Key findings will be highlighted with short engaging videos shared on social media. An event will be hosted inviting young people, academics, health practitioners, charities and policy makers to discuss ways to improve adolescent sleep and demonstrate its crucial importance.

## Supporting information

S1 Checklist (DOCX)
